# Purification of Indole Contained in Wash Oil by Combination of Extraction and Crystallization (Part 1: Recovery and Concentration of Indole Contained in Wash Oil by Solvent Extraction)

**DOI:** 10.3390/molecules27165331

**Published:** 2022-08-21

**Authors:** Su Jin Kim

**Affiliations:** Department of Chemical Engineering, Chungwoon University, Incheon 22100, Korea; sujkim@chungwoon.ac.kr

**Keywords:** coal tar, wash oil, nitrogen-containing compounds, indole concentration, extraction-re-extraction combination

## Abstract

For the purpose of determining the high-purity purification of indole (*IN*) contained in wash oil in concentrations of 5.75%, this study, first of all, investigated the concentration of *IN* contained in wash oil using a combination of methanol extraction to recover *IN* in the wash oil, and n-hexane re-extraction to concentrate *IN* present in the extract phase, recovered through methanol extraction. In order to examine the effect of each operation on the recovery and concentration of *IN* contained in the wash oil, batch 1-stage and batch co-current 5-stage distribution equilibrium was performed simultaneously. As 5-stage equilibrium extraction and re-extraction progressed, the recovery rate of *IN* decreased to about 79.1%, while *IN* composition in the raffinate phase recovered by re-extraction was highly concentrated to about 73.3%. From the high recovery rate and composition of *IN* obtained through this study, we confirmed that the combination examined by this study was one of the very useful combinations for the concentration of *IN* present in the wash oil. Furthermore, we reviewed the recovery and concentration process of *IN* contained in the wash oil using the experiment results of the extraction and the re-extraction obtained from this study.

## 1. Introduction

Wash oil, which is a mixture oil of approximately 10–30 compounds, contains many valuable nitrogen-containing compounds (*NCs*), such as quinoline (*QU*), iso-quinoline, *IN*, and quinaldine. Among them, *IN* has been recognized as an intermediate for the productions of essential amino acids, such as tryptophan, alkaloid-based medicines, and perfumes, such as jasmine and neroli oil [1,2,3,4,5,6]. On the other hand, the *NCs* mentioned above are undesirable impurities in the wash oil because they contribute to air pollution and have unpleasant odors. At the present time, *QU* and the mixtures containing more than 0.1% *QU* are classified as hazardous chemicals (carcinogens, skin irritants, etc.). Therefore, wash oil containing about 1.38% *QU* is a hazardous chemical, so there are many restrictions on transportation and handling, etc. For the above reasons, the improvement of the quality of wash oil according to the reduction in *NC* and the review of the purification of useful components, such as *IN* using the crude *NC* mixture recovered through the quality improvement process, are considered to be very meaningful from the viewpoint of recycling of resources [1].

The composition of *IN* contained in wash oil varies depending on the manufacture company (about 3–7%), but the wash oil used in this study contained about 5.75% *IN*. As shown in Table 1, it can be seen that the boiling points of the compounds included in the washing oil are very close, so it is very difficult to separate each compound by distillation. It is believed that a multi-step procedure should be required to separate and purify of *IN* from the wash oil. The separation and purification of *IN* contained in the wash oil is thought to require the following multi-step procedure: (i) the crude separation of *IN* containing in the wash oil through the solvent extraction operation etc., (ii) the concentration of *IN* containing in the extract through a distillation operation etc., and (iii) the purification of *IN* contained in *IN*-enriched distillate through a crystallization operation etc.

Up to the present, the crude separation of *IN* from the coal tar fraction recovered through distillation of the coal tar was investigated via separation operations, such as extraction using ionic liquids [7,8,9], supercritical extraction [10,11], liquid membrane permeation [4], azeotropic distillation, and traditional extraction methods [1,2,3,5,6,12] using various organic solvents, such as methanol, ethanol, formamide, etc. The purification of *IN* from *IN*-enriched distillate recovered through distillation of the extract was also investigated by an operation, such as inclusion complexation [13,14], adsorption [15], and crystallization [3,16]. 

In a previous study [3], we investigated the concentration of *IN* by the combination of extraction–distillation using a model coal tar fraction. A distillate containing about 54.3% *IN* was obtained by batch distillation of the extract phase, which was recovered by batch equilibrium extraction using an aqueous formamide solution as a solvent. Considering the results of a previous study, the low *IN* composition of the distillate and the high boiling point of formamide (483 K) used as an extraction solvent were raised as one problem.

In this study, the high concentration of *IN* contained in wash oil was experimentally investigated by a combination of extraction using an aqueous methanol solution, which makes it relatively easy to recover the solvent, as an extraction solvent, and re-extraction using n-hexane as a re-extraction solvent.

## 2. Materials and Experimental Methods

### 2.1. Materials

Wash oil, the feed of this study, was provided by OCI Company Ltd. (Seoul, Korea). Naphthalene (*NA*, 99% purity), *QU* (98% purity), *IN* (≥99% purity), 2-methylnaphthalene (2*MNA*, 97% purity), 1-methylnaphthalene (1*MNA*, ≥95% purity), and biphenyl (*BP*, ≥99.5% purity), which were used GC identification or quantification of the constitutive components presenting in the wash oil, were purchased from Sigma Aldrich, Seoul, Korea. Methanol (≥99.5% purity) and n-hexane (≥99% purity), used as the solvent of each operation in this study, were also purchased from Sigma-Aldrich, Seoul, Korea. In this study, the above-mentioned eight kinds of commercial reagents were used without further purification.

### 2.2. Experimental Method

**Figure 1:**
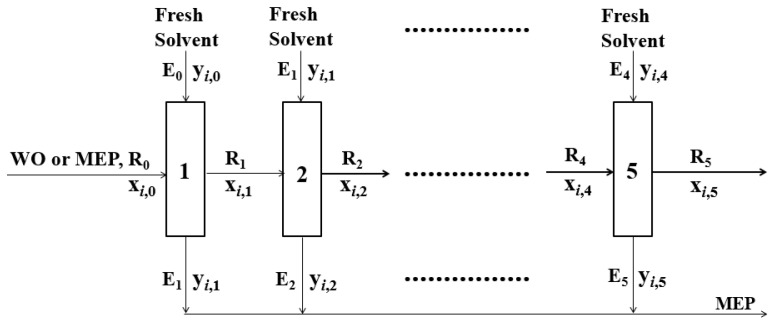
Schematic diagram for equilibrium extraction and re-extraction methods of a batch co-current 5-stage [1]. Abbreviations are as follows: R, raffinate phase; E, extract phase; WO, wash oil; MEP, mixed extract phase; *i*, component *i*; 1, 2, 3, 4, 5, number of equilibrium extraction or re-extraction.

A schematic diagram for extraction and re-extraction methods of a batch co-current 5-stage is presented in Figure 1 [1].

#### 2.2.1. Batch Equilibrium Extraction

To make a solvent of a certain concentration, methanol was mixed with tap water. A 1 L Erlenmeyer flask containing a certain amount of the feed (R_0_—wash oil, R_1_−R_4_—raffinate phases recovered from extraction run of each stage) and the fresh solvent (E_0_–E_4_—aqueous methanol solution) were placed in a shaking water bath maintained at the experimental temperature and vibrated for a certain time to reach a liquid–liquid equilibrium. After reaching the equilibrium, the mixture was settled for an aliquot of time, and the raffinate phase and the extract phase were separated using a 1 L separatory funnel, and then the mass of each phase was measured. The separated raffinate phase and a fresh solvent were used as the feed and a solvent of the next stage, respectively. The raffinate phases and the extract phases recovered from the equilibrium extraction run of each stage were analyzed by adding a small amount of acetone, and their compositions were determined. The analysis of two phases was carried out using a gas chromatograph (GC, Hewlett Packard Co., Houston, TX, USA, HP 6890), with a capillary column, HP-1 (60 mL, 0.32 mm I.D.) and equipped with a flame ionization detector (FID). The analysis conditions of the samples were as follows: carrier gas, N_2_; volume flow rate, 1 mL/min; injection temperature, 523 K; sample volume, 1 µL; splitting ratio, 0.025; column temperature, maintained at 383 K for 3 min, then increased at a rate of 5 K/min to 523 K, then 14 K/min to 593 K; detector temperature was set to 593 K [1].

#### 2.2.2. Batch Equilibrium Re-Extraction

A 1 L Erlenmeyer flask containing a certain amount of the feed (R_0_—a mixed extract phase, which is a mixture of each extract phase recovered through methanol equilibrium extraction of batch co-current 5-stage, R_1_–R_4_—raffinate phases recovered through the re-extraction run of each stage) and the fresh solvent (E_0_–E_4_—n-hexane) were placed in a shaking water bath maintained at the experimental temperature and vibrated for a certain time to reach a liquid–liquid equilibrium. After that, the experimental method, the GC used, and the GC analysis conditions of the samples are the same as those of methanol extraction described above.

### 2.3. Material System and Experimental Conditions

The material system and the experimental conditions according to each operation are summarized in Table 2.

#### 2.3.1. Batch Equilibrium Extraction

Wash oil and the raffinate phases recovered from each stage run were used as the feed, and an aqueous methanol solution was used as the fresh solvent. The operating temperature (T) and the volume of fresh solvent added to each stage (E_0_−E_4_) are kept constant, and the liquid–liquid contact time (t), the number of equilibrium extraction (n), the volume fraction of water in the solvent in the initial state (y_w,0_), and the volume fraction of the fresh solvent to feed in the initial state (E_0_/R_0_) are changed.

#### 2.3.2. Batch Equilibrium Re-Extraction

The mixed extraction phase, which is a mixture of each extract phase recovered through a methanol extraction of n = 1–5, and each raffinate phase recovered in each stage of equilibrium re-extraction run using the above-mentioned mixed extraction phase were used as the feed of the re-extraction operation. Here, t, E_0_–E_4_, and T were kept constant, and n and E_0_/R_0_ were changed.

## 3. Results and Discussion

### 3.1. Definition Equation

Here, m*_i_*_,n_, which is the distribution coefficient of the component *i* obtained by the extraction or re-extraction of the nth stage contact run, is defined as follows:

m*_i_*_,n_ = y*_i_*_,n_/x*_i_*_,n_ (n = 1–5) (1)

where y*_i_*_,__n_ and x*_i_*_,__n_ denote the mass fraction of component *i* in the extract phase and the raffinate phase recovered from the extraction or re-extraction of the nth stage contact run, respectively.

Here, Y*_i_*_,n_, which is the yield of component *i* obtained from the extraction or re-extraction of the nth stage, is defined as follows:

(2) $$Y_{i,n}=(\sum_{n=1}^{5}E_{n}\times y_{i,n})/(R_{0}\times x_{i,0})\times 100\%\;(n=1–5)$$

where R_0_ and x*_i_*_,__0_, respectively, represent the mass of the feed put into n = 1 contact run of the extraction or re-extraction, and the mass fraction of component *i* in the feed. Additionally, E_n_ and y*_i_*_,__n_ denote the mass of the extract phase and the mass fraction of component *i* in the extract phase recovered from the extraction or re-extraction of the nth stage contact run, respectively.

Here, (β*_i_*_,2*MNA*_)_n_ or (β*_i_*_,*IN*_)_n_, respectively, which are the selectivity of component *i* in reference to 2*MNA* or *IN* obtained from the extraction or re-extraction of the nth stage contact run respectively, are defined as follows:

(β*_i_*_,2*MNA*_)_n_ = m*_i_*_,n_/m_2*MNA*,n_, (β*_i_*_,*IN*_)_n_ = m*_i_*_,n_/m*_IN_*_,n_ (n = 1–5) (3)

### 3.2. Confirmation of Equilibrium Arrival Time

To confirm the time of reaching the equilibrium, the raffinate phases or the extract phases recovered through the extraction and re-extraction contact (a contact time of 12 h, 24 h, 72 h, and 96 h) between the feed and the fresh solvent were analyzed. The compositions of the raffinate phases and the extract phases recovered through contact for more than 48 h were almost the same, regardless of the contact time and the operation. Therefore, the liquid–liquid contact time was maintained at 72 h in the entire experiment of this study.

### 3.3. Batch Equilibrium Extraction

#### 3.3.1. Gas Chromatogram of Extraction Feed (Wash Oil)

**Figure 2:**
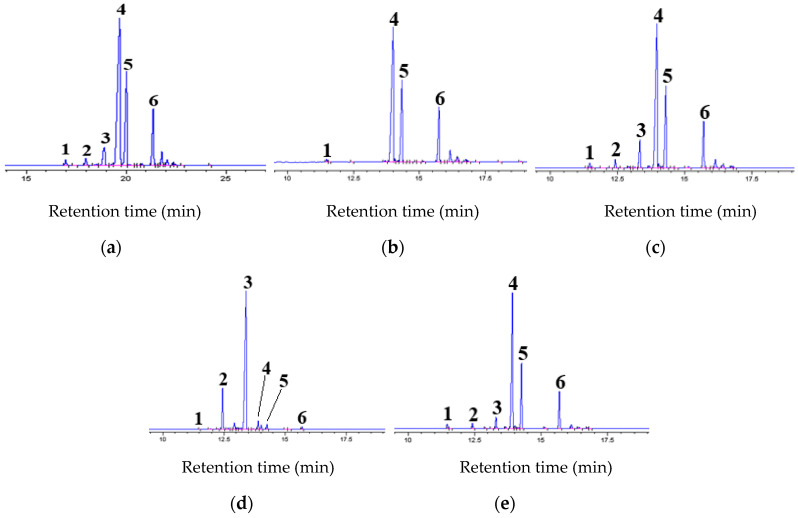
Gas chromatograms of (**a**) extraction feed (wash oil), (**b**) raffinate phase (R_5_) recovered through a methanol extraction run of n = 5, (**c**) re-extraction feed (mixed extract phase ($\overset{5}{\underset{n=1}{\sum }}E_{n})$, which is a mixture of each extract phase recovered through a methanol extraction run of n = 1–5, (**d**) raffinate phase (R_5_) recovered by an n-hexane re-extraction run of n = 5, and (**e**) mixed extract phase ($\overset{5}{\underset{n=1}{\sum }}E_{n}),$ which is a mixture of each extract phase recovered through an n-hexane extraction run of n = 1–5. Extraction experimental conditions are as follows: y_w,0_ = 0.1, E_0_/R_0_ = 1, T = 303 K, n = 1–5, t = 72 h. Re-extraction experimental conditions are as follows: E_0_/R_0_ = 0.5, T = 303 K, n = 1–5, t = 72 h. Peak number 1 was naphthalene (*NA*), 2 was quinolone (*QU*), 3 was indole (*IN*), 4 was 2-methylnaphthalene (2*MNA*), 5 was 1-methylnaphthalene (1*MNA*), and 6 was biphenyl (*BP*).

Figure 2a presents the gas chromatogram of the wash oil used as the extraction feed of this study and the component names of the identified compounds. As mentioned above, they were identified through analysis by adding a small amount of six standard reagents purchased from Sigma-Aldrich Korea. As a result of GC identification, it can be seen that the wash oil contains relatively small amounts of two kinds of *NCs* (*QU*, and *IN*), whereas 2*MNA*, 1*MNA*, and *BP*, excluding *NA* are included in large amounts. The compositions of the 6 compounds quantified in this study are shown in Table 1. The composition of *IN* (peak No. 3), which is the aim component to concentrate in this study, was about 5.75%.

#### 3.3.2. Recovery Performance of IN Contained in Wash Oil

In this study, in order to maximize the recovery of *IN* contained in the wash oil, methanol extraction of the wash oil was performed to investigate the effect of extraction factors and conditions on the recovery of *IN*.

**Figure 3:**
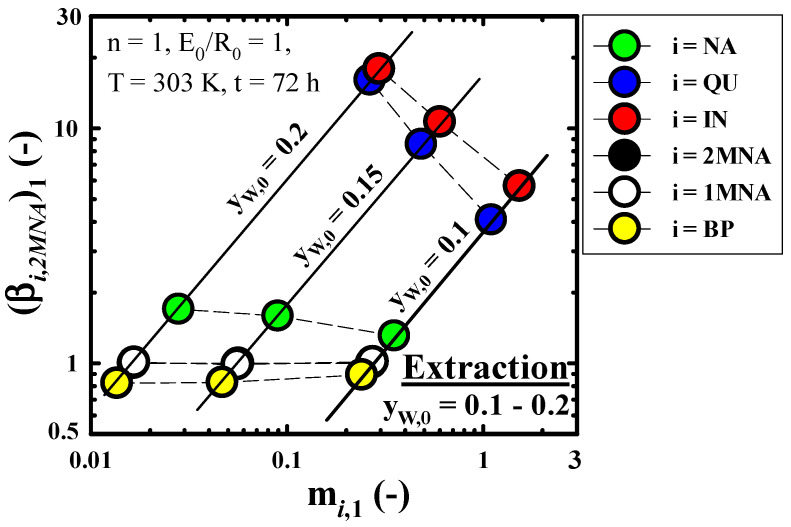
Effect of volume fraction of water in solvent in initial state (y_w,0_) on the distribution coefficient of component *i* (m*_i_*_,__1_) and the selectivity of component *i* in reference to 2*MNA* (β*_i_*_,2*MNA*_)_1_ obtained through a methanol extraction run of n = 1.

Figure 3 shows the effect of y_w,0_ on m*_i_*_,__1_ and (β*_i_*_,2*MNA*_)_1_ obtained in n = 1 of the fixed extraction conditions (n = 1, E_0_/R_0_ = 1, T = 303 K, t = 72 h). The m*_i_*_,1_ of *NCs* (*i* = *QU*, *IN*) with very high polarity were much larger than three kinds of bicyclic aromatic compounds (*BACs*; *i* = *NA*, 1*MN*, 2*MN*) and *Bp*, which were less than the polarity of *NCs*. Increasing y_w,0_ resulted in a decreasing m*_i_*_,1_ of each component because the polarity of the extract phase increased with an increase in its moisture in the extract phase. As can be seen from the definition of (β*_i_*_,2*MNA*_)_1_ and the result of m*_i_*_,1_ of *NCs* mentioned above, (β*_i_*_,2*MNA*_)_1_ of *NCs*, inversely, increased as y_w,0_ increased. This result suggested that the selectivity for the total *NCs* based on the total *BACs* contained in the wash oil could be very high. On the other hand, considering that the values of (β_1*MNA*,2*MNA*_)_1_, (*i* = 1*MNA*), and (β*_BP_*_,2*MNA*_)_1_ (*i* = *BP*) are almost 1, it was found that separation between these components (1*MNA*, 2*MNA*, and *BP*) by methanol extraction was difficult. In y_w,0_ = 0.1−0.2, the ranges of m*_IN_*_,__1_ and (β*_IN_*_,__2*MNA*_)_1_ were about 0.3−1.5 and 5.7−17.9, respectively. The sequence of m*_i_*_,__1_ and (β*_i_*_,__2*MNA*_)_1_ for the each component of this study was *IN* > *QU* > *NA* > 1*MNA* = 2*MNA* > *BP*.

**Figure 4:**
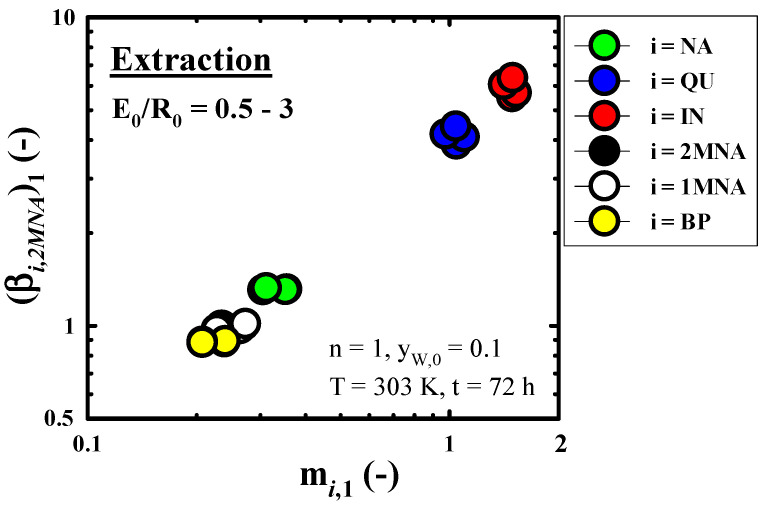
Effect of volume fraction of fresh solvent to feed in initial state (E_0_/R_0_) on the distribution coefficient of component *i* (m*_i_*_,__1_) and the selectivity of component *i* in reference to 2*MNA* (β*_i_*_,2*MNA*_)_1_ obtained through a methanol extraction run of n = 1.

Figure 4 presents the effect of E_0_/R_0_ on m*_i_*_,__1_ and (β*_i_*_,__2*MNA*_)_1_ obtained in n = 1 of the fixed extraction conditions (n = 1, y_w,0_ = 0.1, T = 303 K, t = 72 h). The effect of E_0_/R_0_ on m*_i_*_,1_ and (β*_i_*_,__2*MNA*_)_1_ could not be recognized in all components of this study. The range of E_0_/R_0_ = 0.5−3, m*_IN_*_,__1_ and (β*_IN_*_,__2*MNA*_)_1_ of *i* = *IN*, respectively, showed as about 1.5 and 6.

**Figure 5:**
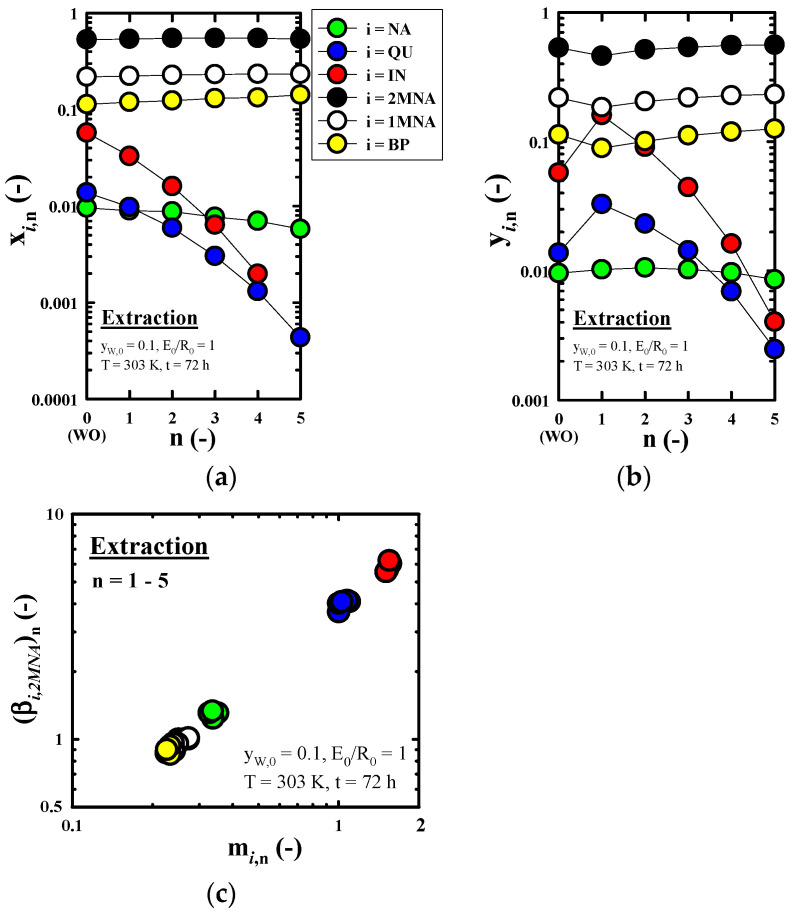
Effect of the number of equilibrium extraction (n) on (**a**) the mass fraction of component *i* in raffinate oil (x*_i_*_,n_, without solvent), (**b**) the mass fraction of component *i* in extract oil (y*_i_*_,n_, without solvent) and (**c**) the distribution coefficient of component *i* (m*_i_*_,n_) and the selectivity of component *i* in reference to 2*MNA* (β*_i_*_,2*MNA*_)_n_ obtained through a methanol extraction run of the nth stage (n = 1–5).

Figure 5a–c, respectively, shows the effect of n on x*_i_*_,__n_ (without solvent), y*_i_*_,__n_ (without solvent), and m*_i_*_,n_ and (β*_i_*_,2*MNA*_)_n_ obtained in the nth stage (n = 1–5) under the fixed extraction conditions (y_w,0_ = 0.1, E_0_/R_0_ = 1, T = 303 K, and t = 72 h) which were selected by considering the recovery rate of *IN* from Figure 3. The n = 0 shown in Figure 5a,b refers to the wash oil used as the feed in this study. As predicted by Figure 3, *NCs* with large polarity are extracted into the extract phase, and it can be seen that the x*_i_*_,__n_ of *NCs* in the raffinate phase decreases rapidly as n increases. On the other hand, the other four components, aside from the *NCs*, showed that x*_i_*_,n_ values were almost constant regardless of n. This is because the compositions of the *NCs* contained in the wash oil are very low compared to three kinds of *BACs* and *BP*, so even if a large amount of the *NCs* with large polarity is extracted as the equilibrium extraction proceed, the composition of *BACs* and *BP* according to the progress of n is not significantly affected. At n = 5, *IN*, which is the target component to recover in this study, was not contained in R_5_. Through this, the effect of recovering *IN* in the wash oil by methanol extraction could be confirmed. It can also be seen from Figure 5b that the y*_i_*_,__n_ of the *NCs* decreases rapidly as the equilibrium extraction progresses, but the y*_i_*_,__n_ of the other four kinds of components presented a tendency to be almost constant. In the ranges of n = 1–4, x*_i_*_,__n_ and y*_i_*_,__n_ of *i* = *IN*, namely x*_IN_*_,__n_ and y*_IN_*_,__n_, showed the ranges of 0.2–3.3% and 1.63−16.0%, respectively. Considering that x*_IN_*_,5_ (*i* = *IN*) did not appear in the raffinate oil recovered at n = 5, it is expected that the quality of the wash oil will be improved by recovering *NC*-free raffinate oil when using a multi-stage column under optimal extraction conditions using methanol. In all components of this study, m*_i_*_,__n_ and (β*_i_*_,__2*MNA*_)_n_ showed almost the same value regardless of n, so the change in the m*_i_*_,__n_ and (β*_i_*_,__2*MNA*_)_n_ according to n could not be recognized. Furthermore, (β*_IN_*_,__2*MNA*_)_n_ was about 6, regardless of n.

Figure 2b,c, respectively, show the gas chromatograms of the raffinate phase (R_5_) recovered through a methanol extraction of n = 5, and a mixed extract phase ($\overset{5}{\underset{n=1}{\sum }}E_{n})$ which mixed each extract phase recovered through a methanol extraction of n = 1−5. When the gas chromatogram of R_5_ in Figure 2b was compared with that of the wash oil in Figure 2a, the peaks of *NCs* (peak numbers 2 and 3) did not appear in the R_5_, and the peak heights of the other compounds was almost the same as those of the wash oil. From the gas chromatogram of the mixed extract phase of Figure 2c, we could see that the peak height of each *NC* was slightly different from that of the wash oil. Two kinds of *NCs* were extracted with the progress of n, and the peak height of each *NC* slightly increased, but the peak heights of other compounds were almost the same.

### 3.4. Batch Equilibrium Re-Extraction

#### 3.4.1. Gas Chromatogram of Re-Extraction Feed (Mixed Extract Phase)

The mixed extract phase in Figure 2c mentioned above was used as the feed for re-extraction in this study. The compositions of six kinds of compounds quantified in this study are shown in Table 1. The composition of the feed of *IN*, the target component for concentration through the re-extraction of this study, was about 1.08%.

#### 3.4.2. Concentration Performance of *IN* Contained in Mixed Extract Phase

In order to concentrate the *IN* contained in the mixed extraction phase recovered by methanol extraction, this study performed a re-extraction operation using n-hexane as a solvent to examine the effect of re-extraction operation factors and conditions on the concentration of *IN*.

**Figure 6:**
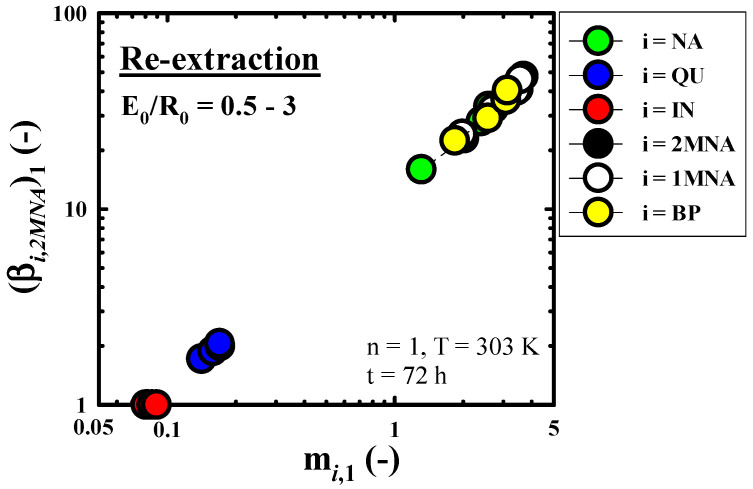
Effect of the volume fraction of fresh solvent to feed in the initial state (E_0_/R_0_) on the distribution coefficient of component *i* (m*_i_*_,__1_) and the selectivity of component *i* in reference to 2*MNA* (β*_i_*_,2*MNA*_)_1_ obtained through a n-hexane re-extraction run of n = 1.

Figure 6 presents the effect of E_0_/R_0_ on m*_i_*_,1_ and (β*_i_*_,*IN*_)_1_ (based on *IN*) obtained in a n = 1 re-extraction run under the fixed re-extraction conditions (n = 1, T = 303 K, t = 72 h). In contrast to the above-mentioned methanol extraction, we found that the m*_i_*_,1_ and (β*_i_*_,*IN*_)_1_ of three kinds of *BACs* (*NA*, 2*MNA*, and 1*MNA*) and *BP* show significantly larger values than those of two kinds of *NCs* (*QU* and *IN*), because *BACs* and *BP* are lipophilic substances with lower polarity compared to *NCs*. In recovering *NCs* in the wash oil, the more difficult the components were to extract from the wash oil using the extraction solvent (an aqueous methanol solution), the easier it was to perform re-extraction. Therefore, contrary to the result of the above-mentioned equilibrium extraction operation of the wash oil, the m*_i_*_,1_ and (β*_i_*_,*IN*_)_1_ sequences of each component were 2*MNA* = 1*MNA* > *BP* > *NA* > *QU* > *IN*. Two kinds of *NCs* showed almost constant m*_i_*_,1_ and (β*_i_*_,*IN*_)_1_ regardless of E_0_/R_0_, but the m*_i_*_,1_ and (β*_i_*_,*IN*_)_1_ of other components, except *NCs*, showed a tendency to increase as E_0_/R_0_ increased. In the range of E_0_/R_0_ = 0.5–3, the m*_i_*_,1_ and (β*_i_*_,*IN*_)_1_ of other components, except *NCs*, showed a very high range of 1.3–3.7 and 15.9–47.6, respectively. From the fact that the m*_i_*_,1_ of *IN* showed the smallest value among six components, it was found that *IN* was concentrated in the raffinate phase by re-extraction using n-hexane.

**Figure 7:**
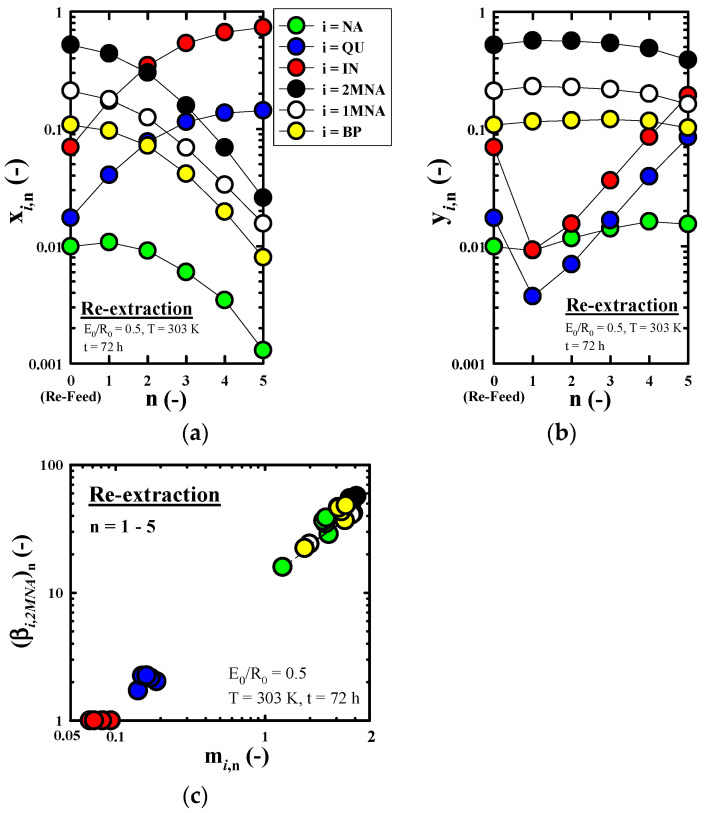
Effect of the number of equilibrium extraction (n) on (**a**) the mass fraction of component *i* in raffinate oil (x*_i_*_,n_, without solvent), (**b**) the mass fraction of component *i* in extract oil (y*_i_*_,n_, without solvent), and (**c**) the distribution coefficient of component *i* (m*_i_*_,n_) and the selectivity of component *i* in reference to 2*MNA* (β*_i_*_,2*MNA*_)_n_, obtained through the n-hexane re-extraction run of the nth stage (n = 1–5).

Figure 7a–c, respectively, show the effect of n on x*_i_*_,n_ (without solvent), y*_i_*_,n_ (without solvent), and the m*_i_*_,n_ and (β*_i_*_,*IN*_)_n_ obtained through the re-extraction run of the nth stage (n = 1–5) under the fixed re-extraction conditions (E_0_/R_0_ = 0.5, T = 303 K, t = 72 h). The n = 0 shown in Figure 7a,b refers to a mixed extract phase which recovered through the methanol extraction used as the feed of re-extraction in this study. As predicted by Figure 6, *NCs* with strong polarity compared to the other four components are concentrated in the raffinate phase, and it can be seen that the x*_i_*_,*n*_ of *NCs* increases rapidly as n increases. On the other hand, the x*_i_*_,*n*_ of the components with strong hydrophobic properties, except *NCs*, decreased rapidly as n increased. At n = 1–5, the x*_IN_*_,n_ (i = *IN*) showed a very high concentration range of about 17.3–73.3%. Through this value, we were able to confirm the effect of the re-extraction operation using n-hexane for the enrichment of *IN*. In Figure 7b, we found that the y*_i_*_,n_ of *NCs* tended to increase sharply as re-extraction proceeded, but the remaining four components showed a nearly constant trend. It can be seen from Figure 7c that m*_i_*_,n_ and (β*_i_*_,*IN*_)_n_ tend to slightly increase as n increases in all components of this study except IN, which is the reference component of selectivity. In the range of n = 1–5, except the *NCs*, the m*_i_*_,n_ and (β*_i_*_,*IN*_)_n_ of the remaining four components showed very high ranges of 1.3−4.1 and 15.9−56.9, respectively.

Figure 2d,e show the gas chromatograms of the raffinate phase (R_5_) recovered through a re-extraction run of n = 5, and a mixed extract phase ($\overset{5}{\underset{n=1}{\sum }}E_{n})$, which is a mixture of each extract phase recovered through a re-extraction run of n = 1–5, respectively. Comparing the gas chromatogram of R_5_ in Figure 2d with that of the feed of the re-extraction operation of Figure 2c, the peak heights of two kinds of *NCs* (peak numbers 2 and 3) were much higher in the re-extraction run, but those of the other compounds were found to be very small. In the gas chromatogram of the extract phase in Figure 2e, we could see that the peak height of each *NC* was slightly different from that of Figure 2c. The *NCs* re-extracted into n-hexane were very small compared to other components, so the peak height of each *NC* decreased, but those of other compounds did not show a significant difference. Through these gas chromatograms, it was possible to reconfirm the effect of re-extraction using n-hexane on the concentration of *IN* presenting in a mixed extract phase recovered through methanol extraction.

### 3.5. Changes of Composition and Yield of IN According as Each Operation

**Figure 8:**
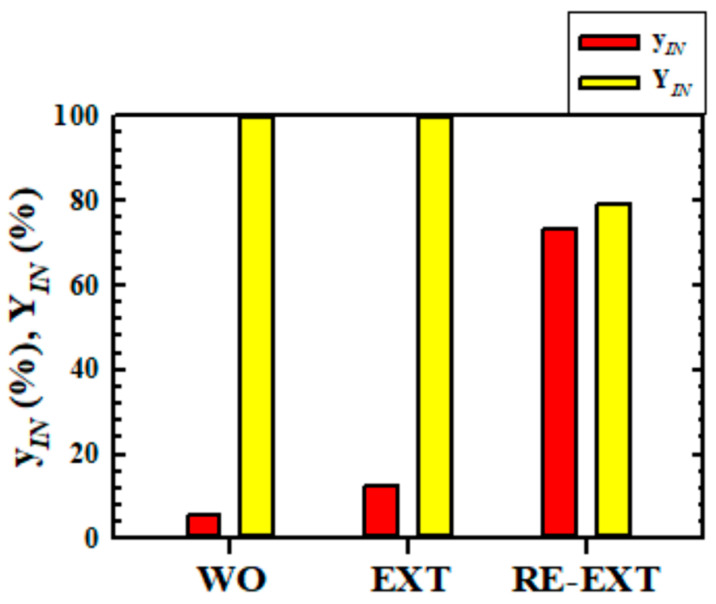
Composition (y*_IN_*) and yield (Y*_IN_*) changes of *IN* in solvent-free raffinate oil obtained at n = 5 for each operation run. Abbreviations are as follows: WO, wash oil; EXT, extraction; RE-EXT, re-extraction.

Figure 8 shows the composition (y*_IN_*) and yield (Y*_IN_*) changes of *IN* in solvent-free raffinate oil obtained at n = 5 for each operation run. The Y*_IN_* in each operation is based on the mass of *IN* contained in the wash oil, and this was calculated by Equation (2). As the extraction and the re-extraction operation progresses, y*_IN_* rapidly increased. The y*_IN_* in the wash oil used as the feed of this study was about 5.75%, but it was concentrated to about 73.3% as methanol extraction and n-hexane re-extraction proceeded. In the extraction operation, 100% of *IN* was recovered, but the loss of Y*_IN_* occurred due to the progress of the re-extraction operation, and about 79.1% of *IN* was recovered.

When comprehensively considering the results of this study, as described above, the combination of methanol extraction and n-hexane re-extraction is expected to be one of the very useful combinations in recovering and concentrating *IN* from wash oil.

### 3.6. Recovery and Concentration Process of IN Containing in Wash Oil

**Figure 9:**
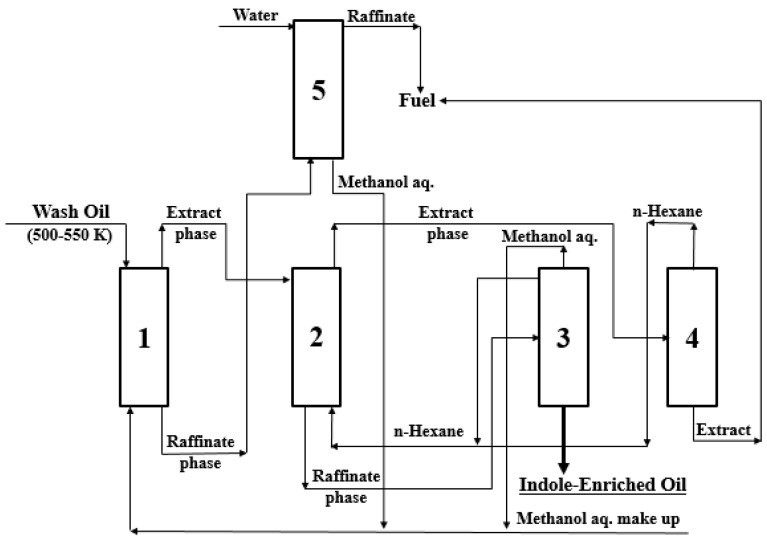
Recovery and concentration process of indole from wash oil. Column number 1 is the extraction column, 2 is the re-extraction column, 3 and 4 are the distillation columns, and 5 is the washing column.

The recovery and concentration process of *IN* from wash oil was investigated using the experimental results obtained from the extraction and the re-extraction of this study. As shown in Figure 9, the process suggested in this study was composed of one extraction column, one re-extraction column, one washing column, and two distillation columns. Column 1 and 2, respectively, are an extraction column for recovering *IN* from the wash oil, and a re-extraction column for concentrating *IN* containing in the extract phase, which is upstream of column 1. Column 3 and 4, respectively, are a distillation column to recover concentrated *IN* oil from the raffinate phase, which is a downstream of column 2, and a distillation column to recover the extract oil from the extract phase, which is an upstream of column 2. Column 5 is a washing column for removing methanol contained in the raffinate phase, which is a downstream of column 1.

## 4. Conclusions

To develop a simpler and more efficient novel process for the high-purity purification of *IN* contained in the wash oil, this study, first of all, examined the concentration of *IN* present in the wash oil. For this examination, this study carried out an equilibrium methanol extraction to recover *IN* in the wash oil and an equilibrium n-hexane re-extraction to concentrate *IN* in the extract phase recovered through an equilibrium methanol extraction. The combination of the methanol extraction and n-hexane re-extraction applied in this study showed very effective results for the concentration of *IN* in the wash oil, so it is expected to be one of the efficient combinations for determining the concentration of *IN* in wash oil. In the next paper, we will report the results of examining the high-purity purification of *IN* through solute crystallization, using the 73.3% *IN*-containing raffinate phase recovered from the re-extraction operation of this study as the feed.

## Figures and Tables

**Table 1:** Physical property and composition of compound contained in the feed used in each operation.

Compound	Physical Property		Composition (wt%)	
	b.p.*[K]	m.p.**[K]	Extraction	Re-Extraction
Naphthalene (*NA*, C_10_H_8_)	491	351–353	0.960	0.128
Quinoline (*QU*, C_9_H_7_N)	511	257	1.379	0.270
Indole (*IN*, C_8_H_7_N)	526	325	5.753	1.075
2-Methylnaphthalene (2*MNA*, C_11_H_10_)	514–515	307–309	53.251	7.198
1-Methylnaphthalene (1*MNA*, C_11_H_10_)	513–516	251	21.854	2.785
Biphenyl (*BP*, C_12_H_10_)	528	342	11.339	1.404
Others			5.464	87.140 (with solvent)

* boiling point, ** melting point.

**Table 2:** Material system and experimental conditions of each operation.

Extraction		Re-Extraction	
Feed: Wash Oil, Raffinate Phase *		Feed: Mixed Extract Phase **, Raffinate Phase ***	
Solvent: aqueous methanol solution		Solvent: n-hexane	
Liquid-liquid contact time, t (h)	12–96	Liquid-liquid contact time, t (h)	72
Number of equilibrium extraction, n (-)	1–5	Number of equilibrium extraction, n (-)	1–5
Operating temperature, T (K)	303	Operating temperature, T (K)	303
Volume of fresh solvent added to each stage (E_0_−E_4_), (mL)	320	Volume of fresh solvent added toeach stage (E_0_−E_4_), (mL)	320
Volume fraction of water in solvent in initial state, y_w,0_ (-)	0.1–0.2	Volume ratio of fresh solvent to feed in initial state, E_0_/R_0_ (-)	0.5–3
Volume ratio of fresh solvent to feed in initial state, E_0_/R_0_ (-)	0.5–3		

* raffinate phase recovered through extraction run of each stage (n = 1–5), ** mixed extract phase ($\overset{5}{\underset{n=1}{\sum }}E_{n})$, which is a mixture of each extract phase recovered through extraction run of each stage (n = 1–5), *** raffinate phase recovered through re-extraction run of each stage (n = 1–5).

## Data Availability

The data presented in this study are available within the article (tables and figures).

## Sample Availability

Samples of the compounds are available from the authors.
